# Do COX-2 inhibitors provide additional pain relief and anti-inflammatory effects in patients with rheumatoid arthritis who are on biological disease-modifying anti-rheumatic drugs and/or corticosteroids? Post-hoc analyses from a randomized clinical trial with etoricoxib

**DOI:** 10.1186/s12891-015-0468-7

**Published:** 2015-02-13

**Authors:** Tore K Kvien, Maria Greenwald, Paul M Peloso, Hongwei Wang, Anish Mehta, Arnold Gammaitoni

**Affiliations:** Department of Rheumatology, Diakonhjemmet Hospital, Box 23, Vinderen N-0319 Oslo, Norway; Desert Medical Advances, Palm Desert, CA USA; Clinical Research, Merck & Co., Inc., Kenilworth, NJ USA

**Keywords:** bDMARDS, Rheumatoid arthritis, Analgesics, Corticosteroids, Pain, COX-2 inhibitors

## Abstract

**Background:**

Our objective was to evaluate the effect of background biological disease-modifying anti-rheumatic drugs (bDMARDs) and/or corticosteroids (CS) on response to nonsteroidal anti-inflammatory drugs (NSAIDs) in rheumatoid arthritis (RA) patients.

**Methods:**

The following efficacy endpoints were evaluated using time-weighted change from baseline in a 12-week, randomized controlled clinical trial with etoricoxib: Patient Global Assessment of Pain, Swollen Joint Count, Tender Joint Count, Health Assessment Questionnaire. The following three treatment groups were evaluated: placebo, pooled etoricoxib 10/30/60 mg, and etoricoxib 90 mg. Screening values, values post flare, as well as changes after treatment were analyzed.

**Results:**

Of the 1014 patients screened, 761 were randomized; 50% were on no background bDMARDs and/or CS therapy, 23% used bDMARDs, 34% used CS, and 8% used both bDMARDs and CS. It was demonstrated that RA patients on bDMARDs or CS had similar pain levels at screening as patients without this co-medication. They experienced flare upon NSAID withdrawal and demonstrated dose-dependent pain improvement with etoricoxib.

**Conclusion:**

These results support that RA patients receiving bDMARDs or CS may still require the use of concomitant analgesics to treat pain. Clinicians should continue to monitor and treat pain even after initiating a bDMARD and/or CS.

**Trial Registration:**

[clinicaltrials.gov; NCT00264147]

## Background

A majority of patients with rheumatoid arthritis (RA) list pain as a priority for improvement [1,2]. Advances in therapeutics (i.e., corticosteroids [CS], synthetic disease-modifying antirheumatic drugs (sDMARDs), and biological DMARDs (bDMARDs) have demonstrated efficacy in reducing inflammation and controlling joint damage in patients with RA [3-5]. However, a building consensus in pain research suggests that chronic persistent pain may result in both peripheral and central nervous system plasticity, establishing a parallel disease process, and that chronic pain, regardless of etiology, should itself be considered a disease [6]. Moreover, recent research has suggested that RA patients can continue to experience significant levels of pain even when underlying disease markers of RA (i.e. DAS-28) are controlled by modern therapeutic regimens [6,7].

If nonsteroidal anti-inflammatory drugs (NSAIDs) have any additive role in RA beyond the use of bDMARDs and CS, it would be predicted that three relationships should hold true: (1) patients with RA and on bDMARDs and/or CS who stop their NSAIDs should have the same degree of flare in RA symptoms as those who are not on such co-medication; ( 2) those on bDMARDs and/or CS should experience the same degree of response with the addition of another NSAID; and (3) dose–response relationships should be similar in patients on versus not on bDMARDs and/or CS. The current report addresses these relationships in *post-hoc* analyses from a primary dose-range-finding clinical trial with etoricoxib, a COX-2 selective NSAID, in RA patients.

## Methods

### Study design and patients

These *post-hoc* analyses are based on a randomized, placebo-controlled, double-blind, multicenter, parallel-group, 5-arm, 12-week trial of etoricoxib (Sponsor protocol # 086, Clinical Trials Registry # NCT00264147) [8]. The study was conducted at 90 sites in four countries (United States, Canada, Colombia, and Switzerland) following approval by local Independent Ethics Committees or Investigational Review Boards, and it was conducted in accordance with Good Clinical Practice principles. The following Institutional Review Boards and Independent Ethics Committees approved the study: College of Physicians and Surgeons of Alberta Research Ethics Review Committee; Ottawa Hospital Research Ethics Board; Institutional Review Board (IRB) of Institutional Review Board Services; Biomedical Research Ethics Board University of Manitoba; Western Institutional Review Board; University of Louisville Human Subjects Protection Program Office; Gundersen Lutheran Ltd. Human Subjects; University of North Texas Health Science Center at Forth Worth Committee; Kantonale Ethikkommission des Kantons Graubünden; Comité de Etica de la Fundación Instituto de Reumatologia Fernando Chalem. Before enrollment, all patients provided written informed consent. Eligible patients were ≥18 years of age and had a clinical diagnosis of RA according to the ARA 1987 revised criteria ≥6 months before enrollment [8]. Patients who flared following withdrawal of stable prestudy NSAIDs were randomized in a 1:1:1:1:1 ratio to placebo, or one of four doses of etoricoxib: 10 mg, 30 mg, 60 mg, or 90 mg daily.

### Endpoints and analyses according to use of bDMARDs and CS

In order to ensure that the findings were generalizable across endpoints, four responsive study endpoints were used that included physician and patient measures: 100 mm pain visual analogue scale (VAS, range 0–100, 100 = worst pain); swollen joint count (out of 66 joints, 66-SJC); tender joint count (out of 68 Joints, 68-TJC); and health assessment questionnaire (HAQ) score (range 0–3, 3 = worst health).

Three subpopulations were evaluated based on the dose–response relationships established in the primary trial analysis: patients on the labeled dose of etoricoxib in RA (90 mg), those on other doses of etoricoxib (10-, 30-, and 60-mg groups combined for these analyses), and patients on placebo. Each of these three groups was further considered based on four possible combinations of bDMARDs and/or CS use (no bDMARD or CS, bDMARD alone, CS alone, or both bDMARD and CS). Those on DMARDs and CS before study entry were continued on the same doses throughout the trial.

### Statistical analysis

The primary population for efficacy analyses was all randomized patients who received ≥1 dose of study medication and had valid baseline and ≥1 on-treatment measurement. Summary statistics for efficacy endpoints were reported by treatment and concomitant medication usage status. Least square means with 95% confidence intervals (CIs) of time-weighted changes from baseline over 12 weeks were generated from an ANCOVA model with terms for baseline parameter, treatment, concomitant medication status, and its interaction with treatment. Due to the limited number of patients in certain strata, the results are mainly for descriptive purposes and should be interpreted accordingly. Our study hypothesis was that etoricoxib would provide similar benefit across the four study endpoints evaluated, independent of the use of biological or corticosteroid co-medication. However, these *post-hoc* analyses were not powered for non-inferiority between groups.

## Results

### Patient characteristics

Baseline demographics were reported in the primary publication for this study [8]. The bDMARDs used in this study included the following: etanercept (n = 68), adalimumab (n = 64), and infliximab (n = 41). Although bDMARDs or CS therapy was used in 23% and 34% of patients, respectively, the subgroup of patients on both agents was small (8%).

Concomitant sDMARDs included methotrexate, sulfasalazine, hydroxycholoquine, gold salts, and leflunomide. Forty percent of patients were taking sDMARDs without bDMARDs or CS, while 19% were not taking sDMARDs, bDMARDs, or CS.

### Screening and baseline values

Screening values (i.e., before randomization and withdrawal of NSAIDs) of the four endpoints were similar across the four subgroups (Table 1). A large pain flare was demonstrated across all subgroups, independently of background RA treatment with bDMARD and/or CS. Increases in tender and swollen joint counts and HAQ-scores were also observed across all four subgroups.

**Table 1:** **Screening means and baseline means (post flare) for pain, swollen and tender joint counts and HAQ scores by treatment groups and by concomitant therapy subgroups**

	**Placebo**	**Etoricoxib 10/30/60 mg**	**Etoricoxib 90 mg**
	**Screening/Baseline**	**Screening/Baseline**	**Screening/Baseline**
**Pain VAS**			
**Neither**	42.8/73.4	39.8/69.0*	37.22/70.6
**bDMARD**	40.2/71.2	39.7/69.4	36.17/67.8
**CS**	38.9/73.1	42.2/74.9	38.23/72.6
**Both**	40.1/69.9	42.7/71.5	48.50 / 73.8
**66-SJC**			
**Neither**	8.1/16.2	7.3/16.6	7.41/15.8
**bDMARD**	9.8/16.9	10.1/15.3	10.17/19.1
**CS**	7.9/15.5	8.9/19.8	7.19 / 14.5
**Both**	10.2/16.6	11.9/17.2	9.63/18.9
**68-TJC**			
**Neither**	13.7/26.5	13.0/26.4	13.86/25.8
**bDMARD**	12.1/29.6	16.8/31.9	15.86/28.9
**CS**	12.5/26.2	12.8/26.8	14.25/25.4
**Both**	13.8/29.1	14.2/29.6	12.81/28.8
**HAQ-score**			
**Neither**	0.99/1.32	0.89/1.18	0.89/1.14
**bDMARD**	0.86/1.32	1.12/1.45	1.01/1.35
**CS**	1.00/1.28	1.02/1.42	0.97/1.26
**Both**	1.57/1.67	1.10/1.43**	1.45/1.64

bDMARDs = Biological disease-modifying antirheumatic drugs;CS = Corticosteroids;VAS = Visual analogue scale.

66-SJC = Swollen joint count of 66 joints; 68-TJC = Tender joint count of 68 joints; HAQ = Health assessment questionnaire.

*p = 0.050 for difference between baseline values for placebo and etoricoxib 10/30/60; **p = 0.036 for difference between screening values for placebo and etoricoxib (Other than these two instances out of the 96 treatment comparisons that were conducted, there were no consistently observed statistical differences in screening/baseline values).

### Response to etoricoxib

**Figure 1:**
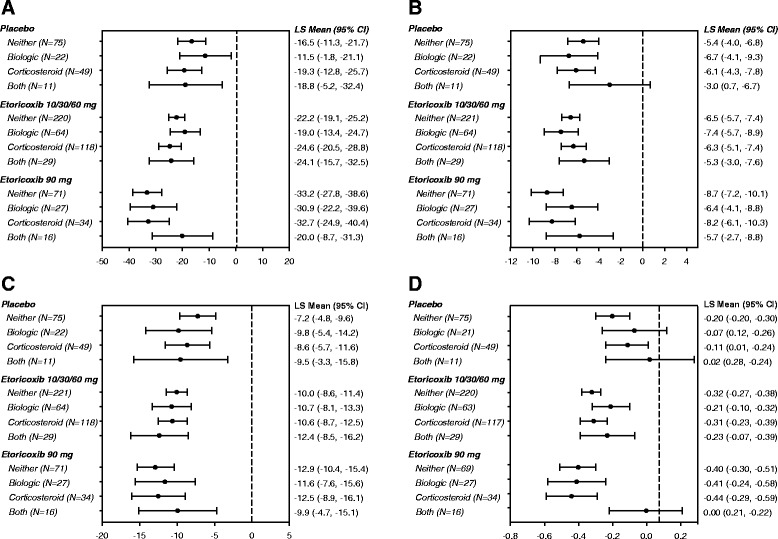
**Least square means (95% CI) change from baseline in efficacy endpoints by trial treatments and concomitant therapy. A)** pain (0–100 mm VAS); **B)** swollen joint count (number of joints); **C)** tender joint count (number of joints); **D)** health assessment questionnaire (points).

Improvements in pain VAS were of similar magnitude across the four subgroups, independent of concomitant treatment with bDMARDs and/or CS (Figure 1). In addition, the data indicated a dose–response relationship from placebo, to the pooled 10-/30-/60-mg dose group, and to the etoricoxib 90-mg dose (Figure 1). However, this dose–response relationship was not evident in the small group (n = 16) using both bDMARDs and CS. Overall, results for the other endpoints (66-SJC, 68-TJC, and HAQ-score) were similar to those observed for pain VAS (Figure 1), suggesting that the improvement was consistent across measures performed by patients and physicians.

## Discussion

No prior studies have examined whether patients with residual pain on bDMARDs benefit from treatment with NSAIDs; these analyses suggest that NSAID treatment in these patients may benefit the patient. Although remission through DMARD use is the focus of treatment in RA [5], pain is still an important issue for patients [1,2]. Thus, from a patient perspective, these data are important for the clinical management of patients with RA, in particular patients with established disease.

Previous studies demonstrate that pain medication usage has persisted even after the introduction of bDMARDs. A study of 24,000 Medicaid patients from 1995 to 2004 showed that, despite increased bDMARDs use, additional pain medication also increased during this time period [9]. Another analysis of healthcare costs in patients on bDMARDs showed high levels of use NSAID and opioid use to control persistent pain [10].

The data reported in these analyses support a role of NSAIDs and selective COX-2 inhibitor therapy, in this case, etoricoxib, as part of a multimodal treatment strategy, alongside treatments that control inflammation in patients who continue to suffer from pain. To the historical views that pain in RA is largely related to joint inflammation or joint damage [11], these data suggest that we need to also add the disease-like plastic changes in the peripheral and central nervous system that contribute to persistent chronic pain beyond the joint pathology [6,7,12]. Importantly, prostaglandins have effects along the entire nociceptive pathway, including the spinal cord, where COX-1 and COX-2 enzymes are expressed [13,14]. The ability of an NSAID to have a central effect through penetration of the blood–brain barrier is variable and depends on various molecular characteristics such as size, lipid solubility, and capacity to bind to plasma proteins [15]. Etoricoxib has demonstrated to be highly bound to plasma proteins [16], and central nervous system penetration has been shown in a study in hip surgery where oral etoricoxib dosing led to meaningful concentrations in cerebrospinal fluid [17]. Thus, NSAIDs such as etoricoxib may have analgesic effects beyond the level of the joint, thereby targeting nervous system plastic responses both in the periphery and, depending on the agent, in the central nervous system as well.

These analyses have limitations related to the nature of *post-hoc* analyses, and additional trials will be needed to confirm these data. The small number of patients in the subgroups limited appropriate sample size calculations and comparative statistical analyses across treatment groups. Particularly, the number of patients in the combined sDMARD and CS group was extremely small. We are not able to make any statements about interactions between sDMARDs and NSAIDs, but most of the patients in all groups were on sDMARDs. Additional limitations are related to the patient population. Average disease duration was 10 years; thus, our results seem to be most relevant for patients with established RA. Additionally, the percentage of patients not on any kind of DMARD or CS was 19%, which may reflect the time period (early 2006) and the multinational nature of this trial [18]. Also, patients may have had high disease activity and were selected after exhibiting a flare of symptoms upon withdrawal of previous NSAID therapy, indicating a proclivity to respond to therapy with etoricoxib while on background therapy. Nonetheless, these data indicate that there is a population of patients with RA who will not obtain adequate pain management with bDMARDs or CS alone.

## Conclusions

Despite these limitations, the consistent trends observed across the four endpoints support that etoricoxib, as an example of an NSAID, provides symptomatic efficacy in RA patients in the presence of concomitant treatment with bDMARDs and/or CS. Given the similarity in the magnitude of flare across the subgroups, the magnitude of response to introduction of etoricoxib and the dose response across the subgroups, these data suggest that NSAIDs, and etoricoxib in particular, have a role in a multimodal treatment strategy to control RA signs and symptoms. However, prescriptions should follow a benefit-risk consideration according to current treatment recommendations.
